# Repair bond strength of bulk fill composites after different adhesion protocols

**DOI:** 10.4317/jced.56129

**Published:** 2019-11-01

**Authors:** Thiago-Clístines de Medeiros, Mariana-Rodrigues de Lima, Stephany-Cimarosti-Figueiredo Bessa, Diana-Ferreira-Gadelha de Araújo, Marília-Regalado Galvão

**Affiliations:** 1 DDS, MSc candidate, School of Dentistry, Federal University of Rio Grande do Norte, Natal, Rio Grande do Norte, Brazil; 2 DDS, MSc, PhD candidate, School of Dentistry, Federal University of Rio Grande do Norte, Natal, Rio Grande do Norte, Brazil; 3 DDS candidate, School of Dentistry, Federal University of Rio Grande do Norte, Natal, Rio Grande do Norte, Brazil; 4 Professor, DDS, MSc, PhD, School of Dentistry, Federal University of Rio Grande do Norte, Natal, Rio Grande do Norte, Brazil

## Abstract

**Background:**

Repairs in composite resin restorations are common procedures in clinical practice. Many surface treatment options have been proposed to improve the adhesion between the old and new composite. The objective of this study was to evaluate the microtensile bond strength of repairs performed on aged bulk fill and conventional composites after different adhesion protocols.

**Material and Methods:**

First, 84 specimens (8x8x4 mm3) of a microhybrid composite and a high-viscosity bulk fill composite were prepared and aged. Afterward, they received a mechanical surface treatment by means of abrasion with a diamond bur, followed by division into six groups according to the adhesion protocol employed: PSA - etching with 35% phosphoric acid + silane + etch-and-rinse adhesive; SA - silane + etch-and-rinse adhesive; PA - etching with 35% phosphoric acid + etch-and-rinse adhesive; A - etch-and-rinse adhesive; PU - 35% phosphoric acid + universal adhesive; and U - universal adhesive. The repairs were performed with a microhybrid composite. Repaired resin blocks were cut into sticks (8x1x1 mm3) and submitted to a microtensile test. Fractured specimens were evaluated to determine the failure pattern (adhesive or cohesive). Data were analyzed by two-way ANOVA.

**Results:**

No statistically significant differences were found in bond strength values among different adhesion protocols and composite types.

**Conclusions:**

The repair bond strength of a bulk fill composite was similar to that found in a conventional composite, with no distinction among adhesion protocols.

** Key words:**Dental restoration repair, composite resins, adhesiveness.

## Introduction

Resin-based composite restorations are routinely performed in clinical dental practice due to the favorable properties of the resinous materials, especially related to aesthetics and the evolution of adhesive systems that allow minimally invasive preparations (1,2). In addition, the durability of these materials has improved in with technological advances in filler particles, monomer matrices, adhesive systems and polymerization devices (3).

Recently, bulk-fill composites have been developed. According to the manufacturers, these composites have a low degree of conversion and can be photoactivated in increments of 4 to 5 mm depth. This characteristic reduces the C-factor, speeding up the process and making the restorative technique less critical by reducing the need for an incremental technique that increases the risk of bubble incorporation and contamination between layers (4).

The technology required for bulk fill composites to be employed in larger increments varies by manufacturer and consists of changes in the chemical composition of these materials, such as employing new types of monomers, more reactive photoinitiators, modified filler particles or larger particles, resulting in increased translucency (4).

Despite the positive characteristics of composite resins, these materials have limitations, so the occurrence of failures is still common, with an incidence of 5% to 45% over a five-year observation period (3). Because of this, practitioners daily face the need to replace or repair restorations due to problems such as pigmentation, marginal leakage, secondary caries and fractured materials (5,6).

Total replacement of restorations is a procedure that weakens the remaining structure, increases the risk of fracture and affects pulp vitality (7); researchers have also determined that restorations with localized defects that were previously repaired showed the same performance after ten years as restorations that had been completely replaced in marginal adaptation, secondary caries, anatomy and color (8). Thus, composite restorations can be considered the treatment of choice for small recurrent caries along the margin, partial staining and fractures (9,10).

During the preparation of a composite restoration, adhesion between increments is facilitated by the presence of an oxygen-inhibited layer that allows a covalent bond to be established between the unpolymerized surface and the newly applied material. However, old restorations do not present this unpolymerized surface layer, hampering the repair process (7,9). In addition, other changes may occur in restorations over time, such as water absorption, chemical degradation and leaching of some components (2,5,11).

To circumvent this issue and improve the union between the remaining restoration and the new restorative material, it is recommended to perform mechanical and/or chemical treatment of the surface to be repaired. Several of these treatments have been proposed in the literature, such as diamond bur abrasion, aluminum oxide sandblasting, silica coating, phosphoric or hydrofluoric acid etching and application of silane and adhesive systems. These treatments aim to increase the surface energy of the material to be repaired and allow better wetting by the adhesive agents (2,5,11). However, since there is a great variation in composition among different composite brands, the materials respond differently to repair techniques; there is still no universally applicable technique (1).

Studies have shown that application of silane prior to the adhesive system provides higher bond strength in repairs of composite restorations (11,12). Silane has two main functional groups: silanol, which attaches to the silica particles of a composite, and the organofunctional group, which binds to methacrylate in the bonding agent. In addition, silane favors the infiltration of the adhesive by increasing surface wettability (5).

More recent self-etching adhesives, called universal adhesives, have silane in their composition and were developed to adhere to different surfaces without the additional application of primers. Therefore, the use of these adhesives may expedite the performance of repair procedures in defective restorations that normally require pretreatment of different substrates, such as dentin, enamel and composite margins (13).

In light of the above facts, this study is justified in carrying out work to evaluate the repair bond strength of bulk fill restorations by means of different adhesion treatments, especially given that it is a recently launched material with increasing incorporation into the clinical practice but few studies in the literature have dealt with this problem.

The aim of this study was to assess the influence of six different types of adhesion protocols on microtensile bond strength of repairs performed on aged bulk fill and microhybrid composite resins. The null hypotheses tested were that there are no differences between the bond strength values of repairs performed on microhybrid and bulk fill composites, and that the protocol of adhesive treatment applied has no influence on the bond strength of these repairs.

## Material and Methods

-Experimental design.

An experimental study was carried out in vitro, having as response variable the repair bond strength expressed in Megapascal (MPa) and as study factors the resin type to be repaired and the adhesive protocols used for repair.

The composition of resinous materials used in this research is detailed in  Table 1.

**Table 1:**
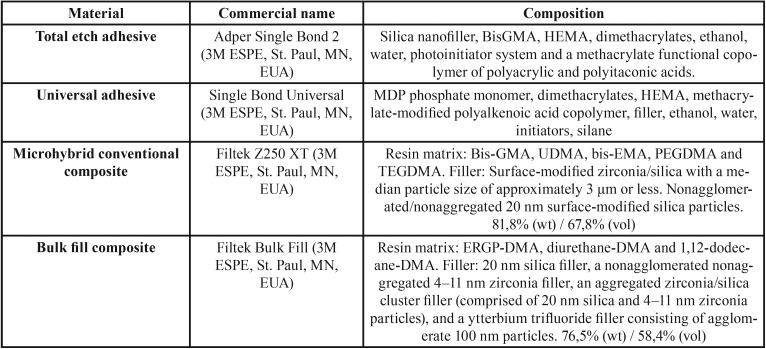
Composition of resinous materials used.

-Preparation of specimens:

A total of 84 specimens were prepared from two different types of resin composites: Z250 XT (3M ESPE, St. Paul, MN, USA) and Filtek Bulk Fill (3M ESPE) in A1 shade. The specimens were prepared using a metal mold made especially for this research, measuring 8x8x4 mm3 (14). The mold was filled with two 2-mm increments for the conventional microhybrid composite resin, and in a single increment of 4 mm for the bulk fill composite, photoactivated for 20 s with a LED light curing unit Optilight Max (Gnatus, Ribeirão Preto, São Paulo, Brazil) with a light power of 1,200 mW/cm2 verified by radiometer. Before polymerization of the last composite layer, it was covered by a polyester strip and a glass slide to obtain a smooth surface. Then the glass slide was removed, keeping the polyester strip in contact with the composite to protect it from the oxygen inhibition layer (11). After removal of the metal mold, the blocks were exposed to an additional photoactivation for 20 s on each unexposed face (9).

The specimens were stored in artificial saliva for 30 days at 37 °C for aging (15).

-Surface treatments:

The two types of composites received mechanical treatment with a fine-grit diamond bur for three seconds, with the burs replaced by new ones after every five blocks treated (2,7,10).

After the mechanical treatment, the specimens were then randomly subdivided in six subgroups according to the adhesion protocol employed:

PSA - 35% phosphoric acid + Silane + etch-and-rinse adhesive (Adper Single Bond 2 - 3M ESPE).

SA - Silane + etch-and-rinse adhesive.

PA - Etching with 35% phosphoric acid + etch-and-rinse adhesive.

A - Etch-and-rinse adhesive.

PU - 35% phosphoric acid + universal adhesive (Single Bond Universal - 3M ESPE); and

U - Universal adhesive.

-Repair:

The specimens were repaired by the addition of two 2-mm increments of Z250 XT microhybrid composite in shade B3 to differentiate them from the original restoration, following the same photoactivation scheme used in the preparation of the substrates.

Repaired composite blocks were stored in distilled water at 37°C for 24 h and then submitted to the microtensile bond strength test (15).

-Microtensile test:

Each of the resin blocks repaired was cut longitudinally on two perpendicular shafts with the aid of a cutting machine under refrigeration with distilled water (Isomet, Buehler, USA), resulting in sticks with a cross-section of approximately 1x1 mm2. Sticks that fractured or detached during the cutting procedure were discarded and not counted in the statistical analysis (2,16).

The sticks were affixed with a cyanoacrylate-based glue (IC-Gel, BSI, Atascadero, CA, USA) to a metal device that was coupled to a semi-universal testing machine (Microtensile OM100, Odeme, Luzerna, SC, Brazil) at a crosshead speed of 0.7 mm/min and load cell of 450 N. The force required for the fracture was recorded in Newtons (N) and divided by the bonding interfacial area (mm²) to express the repair bond strength in Megapascal (Mpa) (11,16).

-Fracture pattern analysis:

The specimens were photographed using a magnifying glass to determinate the fracture pattern (adhesive when the fracture occurred at the adhesive interface, or cohesive when the fracture occurred in the composite substrate).

-Statistical analysis:

The Kolmogorov-Smirnov test was performed to verify the normality of the bond strength values distribution within the various groups. Two-way analysis of variance (ANOVA) at a 95% confidence interval was used to determine the occurrence of significant differences between composite groups, among adhesion protocols and the occurrence of interaction between these variables.

## Results

The results showed no statistically significant difference (*p*> 0.05) among the analyzed treatments for each composite, nor between the different composites for each adhesive protocol ( Table 2).

**Table 2:**
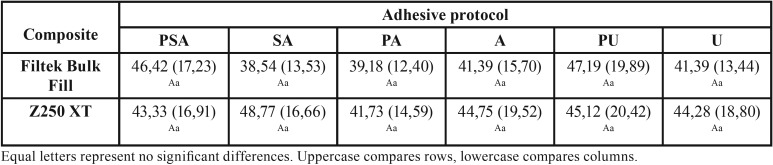
Means and standard deviation of the repair bond strength values (MPa) after different adhesive protocols.

The fracture pattern analysis after the microtensile test showed a predominance of adhesive failures among Z250 XT repairs, except for the PU and U groups. Among Filtek Bulk Fill groups, there was a predominance of adhesive fractures in the groups PSA, A and PU and cohesive fractures in the SA, PA and U groups ( Table 3).

**Table 3:**
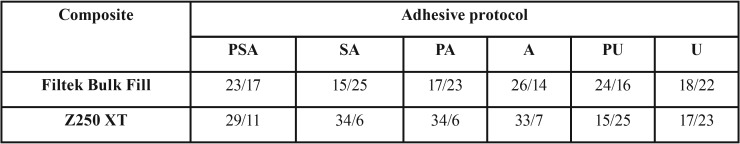
Distribution of the fracture pattern after microtensile test (adhesive/cohesive).

## Discussion

The null hypotheses were accepted because the types of adhesive protocol did not influence the repair bond strength values, as well as the lack of statistically significant difference between the Filtek Bulk Fill and Filtek Z250 XT groups.

The repair bond strength of composites is usually measured by means of the shear bond or microtensile bond strength tests. The shear bond strength test has been criticized for not producing homogeneous stress distribution at the adhesive interface, causing the fracture to start frequently in one of the composites, underestimating the true bond strength value (16). For this reason, the microtensile bond strength test was chosen for this study.

The specimens were aged for 30 days in artificial saliva at 37°C to simulate oral cavity conditions (10,11,15). Water absorption has a negative effect on composite restoration, causing a reduction in resistance to wear, residual monomers and hydrolytic degradation of chemical reactions (17). According to some authors, the highest water absorption occurs in the first week, and in up to two weeks, it is still possible to obtain chemical adhesion of new composite increments due to the presence of free radicals available in the old composite (18).

Due to the reduced capacity to produce chemical reactions in repairs of aged restorations, it is necessary to perform a mechanical surface treatment to produce areas of macro- and micro-retention and increase the exposure of resin matrix and inorganic filler particles. In this research, a diamond bur was employed for roughening because it is a routinely used treatment in clinical practice, as well as its availability, technical simplicity and proven effectiveness in repair procedure research (7,10).

In addition to mechanical treatment, it is necessary to perform chemical treatments by applying intermediate bonding agents, which function by bonding with organic matrix and exposed filler particles, promoting micromechanical retention (19).

According to some authors, the application of phosphoric acid is important in repair procedures because it removes organic contamination and waste left by the mechanical treatment, favoring the reaction between silane and inorganic particles (5).

Regarding silane, discrepant results are found in the literature. Hamano *et al.* (9) concluded that this material did not increase the repair bond strength in comparison to an adhesive alone and should therefore not be used because it could contaminate the enamel or dentin in the process. On the other hand, Staxrud and Dahl (12) observed improvement in repair bond strength – especially in aged specimens – after silane application, either in a separate step or as a constituent of an adhesive. Fornazari *et al.* (20) further claim that an MDP-containing silane can chemically adhere to zirconia (a component of some brands of composites) better than conventional silane.

This study tested the effect of differences in the composition of different adhesive generations. Single Bond Universal contains silane and 10-MDP in its composition; these components can bind chemically to the surface of zirconia, a substance present in some filler particles (13). Another characteristic of universal adhesives is their greater hydrophilia, which could facilitate penetration into the surface of aged restorations that have absorbed water from the oral environment. However, that hydrophilic property can have negative consequences by preventing primer solvents from evaporating before adhesive penetration (12).

Single Bond 2, which also has hydrophilic components, was analyzed in repair research that observed silver nitrate uptake in specimens maintained for six months in water, which did not occur when a solvent-free adhesive was used (21). Celik *et al.* (22) observed no differences between repairs using hydrophilic and hydrophobic adhesives aged by 1,000 cycles of thermocycling, but recognized the need for longer aging analyses.

The results of this research did not show a statistically significant difference among adhesive systems, with or without the prior use of phosphoric acid and/or silane, for the two types of composites repaired. Prior studies show different results in research involving various combinations of treatments and materials. 

As in this study, Fornazari *et al.* (20), found that the application of a universal adhesive (Scotchbond Universal) was as effective as several combinations involving silane and a conventional adhesive (Heliobond). In contrast, Kiomarsi *et al.* (7) evaluated repairs on Z250 composites and observed that Single Bond Universal significantly increased repair bond strength when compared to Adper Single Bond 2 and silane.

Studies involving Clearfil SE Bond, a self-etching adhesive, showed that this material provided greater bond strength than other types of adhesive systems. The authors attributed those results to the presence of 10-MDP (18,23). Clearfil SE Bond, unlike Single Bond Universal, is a two-step system.

The other study factor of this research concerns the different types of aged composite to be repaired: Filtek Bulk Fill and Filtek Z250 XT. Because it is a recently released material, research on bulk fill repair is scarce in the literature, and it is important to evaluate if the inherent differences of this material affect its reparability compared to conventional composites.

The main property that characterizes bulk fill composites is its low polymerization shrinkage stress, which allows the use of layers up to 4 or 5 mm in making restorations. Each manufacturer has its own technologies to obtain this feature, which involve changes in composites’ structure, such as the use of specific monomers and different photoinitiators (4). According to manufacturer, Filtek Bulk Fill contains two new monomers that work together to reduce polymerization stress (24).

According to Mansouri and Zidan (17), materials with lower particle filler concentration and higher resin matrix content are more subject to water absorption. In a study carried out by these authors, the Filtek Bulk Fill composite, which contains less filler content than Z250 XT, showed higher water absorption and lower solubility, although without statistically significant difference between these composites.

In this study, Z250 XT was chosen as a repair material for both substrates because it is a widely used microhybrid composite, and also in order to simulate conditions in which the dentist does not know the material of the original restoration.

In an inverse simulation, a bulk fill composite showed significantly lower bond strength than conventional composites when used as repair material of nanoparticulate composite Filtek Z350 (10). In research involving only Z250, no significant differences were found between different adhesion protocols (14).

The fracture mode analysis showed different results among the materials. In Z250 XT repairs, there was a predominance of adhesive failures when Adper Single Bond 2 was used, according to other research that used this adhesive system (10). On the other hand, the Z250 XT repairs with Single Bond Universal adhesive presented a greater number of cohesive failures, as observed in another study (7). Among the Filtek Bulk Fill repair groups, there was no marked predominance of adhesive or cohesive failure, which may indicate a lower cohesive strength of this material, related to its lower concentration of filler particles (17).

A minimum value for clinically satisfactory repair bond strength has not been established. However, some authors use the composite-to-enamel bond strength, rated between 15 and 30 MPa (2,22). The mean values of all groups evaluated in the present study were considerably higher than that, which also may explain the higher occurrence of cohesive fractures in some groups.

The results of this research provided information of great practical utility to dentists in the use of new materials for repair procedures. It is important, however, that clinical trials are conducted to evaluate the durability of these procedures in function.

Considering the simulated aging and the mechanical surface treatment adopted in this research, repairs of bulk fill composites are feasible and present bond strength comparable to repairs of conventional composites. The different adhesion protocols analyzed resulted in satisfactory and similar bond strength values, giving professionals the opportunity to choose among several options for composite repair procedures.
